# Exercise Oncology and Immuno-Oncology; A (Future) Dynamic Duo

**DOI:** 10.3390/ijms21113816

**Published:** 2020-05-27

**Authors:** Gitte Holmen Olofsson, Agnete Witness Praest Jensen, Manja Idorn, Per thor Straten

**Affiliations:** 1 Department of Oncology, National Center for Cancer Immune Therapy (CCIT-DK), University Hospital Herlev, 2730 Herlev, Denmark; agnete.witness.praest.jensen@regionh.dk; 2 Department of Biomedicine, Faculty of Health, Aarhus University, 8000 Aarhus, Denmark; manja.idorn@biomed.au.dk; 3 Department of Immunology and Microbiology, Faculty of Health and Medical Sciences, University of Copenhagen, 2200 Copenhagen, Denmark

**Keywords:** exercise, physical activity, cancer, immunotherapy, immune system

## Abstract

Recent advances in clinical oncology is based on exploiting the capacity of the immune system to combat cancer: immuno-oncology. Thus, immunotherapy of cancer is now used to treat a variety of malignant diseases. A striking feature is that even patients with late-stage disease may experience curative responses. However, most patients still succumb to disease, and do not benefit from treatment. Exercise has gained attention in clinical oncology and has been used for many years to improve quality of life, as well as to counteract chemotherapy-related complications. However, more recently, exercise has garnered interest, largely due to data from animal studies suggesting a striking therapeutic effect in preclinical cancer models; an effect largely mediated by the immune system. In humans, physical activity is associated with a lower risk for a variety of malignancies, and some data suggest a positive clinical effect for cancer patients. Exercise leads to mobilization of cells of the immune system, resulting in redistribution to different body compartments, and in preclinical models, exercise has been shown to lead to immunological changes in the tumor microenvironment. This suggests that exercise and immunotherapy could have a synergistic effect if combined.

## 1. Recent Breakthroughs in Immunotherapy of Cancer

The treatment of disseminated cancer has been revolutionized by the introduction of immunotherapy. Although several approaches have led to FDA and/or EMA approval, the most widely applied immunotherapy is based on blockade of the PD1/PDL1 interaction. Blocking of this interaction is based on the use of monoclonal antibodies (mAb) specific for either PD1 or PDL1, and thus blocking the inhibitory signaling in cells of the immune system [1]. Several cell types may express the PD1 molecule, most notably T and NK cells. Likewise, several cell types may express the ligand, most notably cancer cells, and cells of the innate immune system [2]. Use of these mAb has been approved in numerous cancer indications, e.g., melanoma, non-small cell lung cancer (NSCLC), head and neck cancer, bladder cancer, and renal cell carcinoma (RCC). Importantly, in some diseases these immunotherapeutic drugs now represent first-line treatment [3]. Another striking feature of these treatments is that even patients in late-stage cancer may experience lasting complete responses, i.e., cure from disease [3].

The therapeutic potential of blocking a single inhibitory pathway is truly impressive and suggests that deeper responses, as well as higher response rates, could potentially be accomplished by combination therapies; with either conventional therapy, radiation or chemotherapy, or with additional immunotherapies. In the last case, combinatorial treatment by targeting PD1 and CTLA-4 has been approved for the treatment of advanced melanoma [4], and very recently FDA approved this combo for treatment of patients with NSCLC [5,6]. Moreover, effector cells of the immune system, T and NK cells, are regulated by the expression of numerous coinhibitory and costimulatory molecules [7]. Numerous clinical trials are ongoing to investigate the effect of combining PD1/PDL1 breach with either an agonistic mAb specific for a costimulatory molecule, or with an additional blocking antibody specific for an inhibitory molecule expressed by T and/or NK cells [8].

Despite the above-mentioned revolution in the management of disseminated cancer, most patients do not respond to treatment, or experience disease progression upon initial response. Moreover, there is an urgent call for characterization of predictive markers, or the development of treatments or modifications that lead to efficacy in a larger fraction of patients. 

## 2. Response Markers in the Tumor Microenvironment (TME)

Clearly, since PDL1 is expressed by cancer cells, the expression of PDL1 has been scrutinized in numerous studies and is indeed used to select patients for treatment in some diseases. However, for most cancers, the expression of PDL1 is not a sufficiently strong marker to indicate response, which implies that most patients are treated irrespective of PDL1 expression, or the use of any other marker for that matter [9].

Despite this, several studies have focused on various aspects of the tumor microenvironment (TME), in the search for traits that could be related to clinical response [9]. In colon cancer patients, the mutational burden has been shown to correlate with response to therapy; treatment is provided if tested positive for microsatellite instability, which is indicative of high mutational load [10]. In this case, it is purported that the mutational burden renders cancer cells more immunogenic due to the expression of a higher fraction of none-self neoantigens derived from gene mutations [11]. On the other hand, some mutations, e.g., beta-catenin, have been shown to lead to the exclusion of T cells from the TME [12].

Tumors are infiltrated by cells of the immune system, i.e., T and NK cells. The term “hot tumor” defines tumors with “many” infiltrating CD8 T cells as opposed to the “cold tumor” which is characterized by the presence of more limited numbers of CD8 T cells [13]. Very early studies described CD8 T cells in melanoma lesions as an independent prognostic marker associated with improved overall survival [14]. Comparable small studies have been conducted in other cancers with similar conclusions, but in recent years a key step forward has been the development of the immunoscore—a scoring system to quantitate CD3 (total T cells) and CD8 (cytotoxic T cells) T cells in a robust and standardized manner [15]. Importantly, the immunoscore has been shown to be able to more accurately predict the overall survival of patients with colorectal cancer, than the conventional TNM system [16]. Even though some infiltrated T cells may remain outside the core of the tumor, the above data demonstrate that T cell infiltration into tumors may synergistically aid immunotherapy with checkpoint inhibitors (CPI). 

Several studies have shown a correlation between response to PD1/PDL1 blockade and the infiltration of T cells. Intuitively, this makes a lot of sense, given the supposed mechanism of action; that tumor-specific T cells in the TME are functionally suppressed via the engagement of PD1 expressed by the T cell, with PDL1 expressed by cancer cells. Additionally, IFN-γ leads to the upregulation of PDL1 by cancer cells, highlighting the link between activated T cells in the TME and expression of PDL1 [2]. Indeed, several studies have shown that a gene signature linked to IFN-γ expression, is correlated with response to anti-PD1 therapy [2].

It should be mentioned that although immunoscore has been shown to correlate with a response in some studies, there are still discrepancies. Patients with very few T cells may respond, and vice versa—patients presenting with hot tumors are by no means guaranteed response to treatment. Several new developments are important in trying to more precisely pinpoint which immunological characteristics can predict response, in addition to i.e., presence of high numbers of T cells, and an IFN-γ gene signature [17]. Nonetheless, the presence of high numbers of T cells, i.e., a hot tumor, is correlated with improved overall survival and response to PD1 blockade [15].

This underscores the important question of why a cold tumor is cold, and whether a cold tumor can be converted to a hot tumor? To the former, it has been demonstrated that loss or downregulation of HLA molecules by cancer cells is associated with limited infiltration of T cells [18]. This means of escape emphasizes the potential of (also) engaging NK cells in immunotherapy of cancer [19]. infiltration of NK cells to tumors is generally poor. However, in some malignancies NK cells may be more abundant and a positive correlation to disease progression has been suggested [19]. Importantly, NK cells may express PD-1 and thus could play a role in response to PD1/PDL1 checkpoint inhibition [20]. Concerning the possible conversion of the cold tumor, several combinatorial treatment approaches are currently being examined to test this notion, including vaccination, adoptive cell transfer, or depletion of suppressive cell subsets [13,17]. Improved numbers of T cells in tumors could be accomplished by means that allow intra-tumor T cells, although few in numbers, to proliferate, or to increase the efficiency of T cell recruitment to the tumor. To the latter point, exercise has been shown to mobilize cells of the immune system, both in man and mouse, and could thus play a role to increase immune cell infiltration to tumors and modulate the tumor microenvironment.

## 3. Preclinical Data from Rodent Animal Models of Cancer and Exercise

Exercise and physical activity in rodent animal models has been associated with a multitude of beneficial effects, including increasing appetite, and lowering adverse effects of therapy, e.g., limiting weight loss (cachexia) and muscle wasting [21,22,23]. This has led to the establishment of exercise programs offered to cancer patients before or during treatment. The main aim is often to increase quality of life, but also to limit cachexia and loss of muscle strength. Based on animal studies, an increasing body of evidence agrees that exercise also has a direct effect on cancer incidence, progression, and metastasis; however, the exact mechanisms remain elusive. 

A variety of preclinical models have been used to try and assess the effect of exercise on cancer progression and outcome. Across a multitude of cancer models (transplantable, chemically induced, or genetic; subcutaneous or orthotopic), these studies have included a wide range of exercise modalities—voluntary or forced, endurance or exhaustive/high-intensity—and most studies report a beneficial effect of exercise. However, some studies have shown the contrary.

Two recent reviews are based on numerous key studies in the field [24,25]. The study by Pedersen et al. summarized 88 studies reporting on physical activity in rodent tumor models performed between 1945 and 2014, in which the animal model, exercise modality, cancer type, and effect was compared [24]. Approximately 60% of studies demonstrated an inhibitory effect on tumor incidence, multiplicity, and growth, and 30% reported no effect. Only 8% reported a tumor-promoting effect of exercise on cancer. Ashcraft and colleagues summarized data on the effect of aerobic exercise on tumor initiation, progression, and metastasis in animal models, and found a total of 53 studies performed between 1974 and 2015 [25]. The main conclusion, however, is that there is a huge degree of heterogeneity in how the studies of exercise oncology are conducted, to the level that it hampers comparison and conclusions to be made.

As an example, the rodent Walker 256 tumor model has been used by several researchers for studies in exercise oncology, but with opposing outcomes. To this end, a study from 1974 [26] reported that while the growth of transplantable intramuscular Walker 256 tumors was similar in exercising and nonexercising rats, exercise increased the number of metastases arising from the primary lesions. On the contrary, studies from de Lima et al. [27,28] and Moreira [29] found that exercise reduced tumor growth of Walker 256 tumors in rats. A crucial difference in the studies, among many, is the exercise modality used. The 1974 study [26] used 15 min forced swimming for 10 days post-tumor inoculation, while the 2008 study employed eight weeks of jump training (four times/week), six weeks prior and two weeks post-tumor inoculation [27]. Alternatively, Moreira and colleagues [29] exercised rats on a treadmill three times/week for eight weeks prior to tumor inoculation, but no exercise was assigned after tumor inoculation. 

Herein lies the crux of the problem. The studies include considerable methodological heterogeneity, which makes it difficult to make any reliable comparison. The authors of both the above-mentioned systematic reviews conclude that the variety of outcomes is a result of poor methodological consistency and recommends methodological and data reporting standards for future preclinical studies in exercise oncology. In addition, in a very recent meta-analysis evaluating the effect of exercise on metastasis, it was impossible to conclude the general pro- or antimetastatic effect of exercise due to the wide methodological heterogeneity [30]. As these methodological limitations have been extensively discussed and reviewed by Pedersen, Ashcraft, and Rincón-Castanedo, we will not go into further detail here. It does, however, highlight that literature in this field should be evaluated with caution. Moreover, studies that do not make attempts to reveal the mechanism of action are also hampered by the associated limited tools to mimic or block the effect of exercise and test in other models. 

Despite the mentioned obvious limitations making direct comparisons between methodologically different studies, there are some quite interesting indications that training regimen and timing of exercise may be important. Exhaustive exercise and extensive high-intensity training have been shown to either promote cancer progression [31] or at best, abolish the tumor limiting effects observed with endurance training [32]. In this respect, data suggest that exhaustive exercise may result in a deeper drop in the lymphocyte level, and suppress the return to baseline for a longer period (than normal endurance training) [33], thus opening an “immunocompromised” window. 

In addition, the timing of exercise the regimen may play a role, at least in rodent models of cancer. While most of the published studies on exercise inhibition of tumor growth have 2–8 weeks’ exercise modality prior to tumor inoculation coupled with continued exercise after tumor challenge, interestingly in some studies animals only exercised prior to tumor inoculation, yet, still observed an effect of exercise [29,34]. We observed a similar growth reducing effect in mice exercised four weeks prior to inoculation with B16-F10 tumors, and no subsequent exercise during tumor challenge [35]. 

On the contrary, many rodent models of exercise fail to show an effect on tumor growth and/or survival if the exercise regimen is first initiated after tumor inoculation [35,36,37,38]. To this end, MacNeil and colleagues [37] observed that exercise prior to tumor inoculation decreased metastasis as discussed above, whereas exercise after tumor inoculation did little on its own. Animals exercising for several weeks prior to tumor inoculation become acclimatized to the equipment and the training situation, allowing for a non-stress-related evaluation of the exercise effect. Exercise and stress are two very different sides of the “fight or flight” response, in the form of epinephrine (EPI) and norepinephrine (NE) [39]. These catecholamines can be released both as neurotransmitters in adrenergic neurons of the sympathetic nervous system, as well as secreted hormones from the adrenal gland. EPI and NE can bind to the same beta-adrenergic receptors at different affinities, thus the downstream responses are different [39]. Where exercise in animal models has primarily been associated with reduced tumor growth, stress responses have been associated with tumor promotion [40,41,42,43]. Thus, if the animals are not acclimatized to the exercise equipment or training situation, introducing animals to exercise regimens after tumor inoculation may carry the bias of introducing a full-blown stress response in the animals, counteracting the antitumor effect of exercise on cancer. Supporting this notion are the few long-term cancer models such as chemically induced liver carcinomas (DEN model) where exercise is introduced after chemical induction, and prior to clinically detectable tumors and yet show a significant impact of exercise [32,35]. It should also be mentioned that a few studies also find an effect of exercise when initiated after tumor inoculation [44,45].

As the tumor models used in most preclinical studies are very fast-growing, a repeated suppression of the immune system by either exhaustive, high-intensity exercise or the stress of adaptation to exercise regimens after inoculation, could explain the unchanged or even promoted tumor progression in these models.

## 4. Mechanisms Behind Exercise-Induced Tumor Growth Control

The bulk of preclinical studies supporting the antitumor effect of exercise have been linked to the multitude of beneficial effects of exercise. During an acute bout of exercise, physical changes occur including increased blood perfusion and vascularization, oxygen consumption, body temperature increases, and exercise hormone secretion, such as catecholamines and myokines (reviewed by Idorn [46]). In trained animals, exercise adaptations comprise systemic alterations with improved immune function, reduced systemic inflammation, and improved metabolic health (increased glucose tolerance and insulin sensitivity) [29]. In a cancer setting, these responses have been associated with intratumoral changes including enhanced blood perfusion, tumor vessel normalization [47], decreased hypoxia, and intratumoral metabolic stress (decreased availability of glucose and glutamine, less production of lactate, etc.) [35,48,49]. In addition, exercise can modulate the tumor microenvironment, making it more permissible for the infiltration of immune cells. To this end, data indicate that exercise can increase the infiltration of antitumor immune cells, thus shifting the microenvironment away from one dominated by immunosuppressive cells. [34,35,45]. 

We reported that exercise decreased tumor incidence in a chemically induced liver carcinoma model as well as a genetic mouse model of melanoma. In addition, tumor size of subcutaneous transplantable, as well as the number of lung “metastases” after tail vein injection of B16-F10 melanoma cells, were reduced by >60% in exercising mice. This effect was associated with the mobilization and redistribution of T and NK cells to the tumor [35]. The effect of exercise mediated by immunological mechanisms has been suggested before [37,48], and has since been corroborated by data showing improved tumor control in exercising animals inoculated with transplantable 4T1 breast tumors [34,45,50].

Thus, Hagar et al. showed that endurance training decelerated 4T1 tumor progression likely facilitated by exercise mediated decrease in FoxP3^+^ Treg cell numbers observed in the tumors of exercising mice, which in turn led to a more favorable CD8/Treg ratio. This effect of exercise was completely abolished in athymic nude mice [34].

Mechanistically, this is unlike our own findings in the B16-F10 melanoma model, where T cells were dispensable for the antitumor effect of exercise (assessed in nude mice); our model was highly dependent on NK cells [35]. This may be due to the immunogenicity of the tumor (B16 being very susceptible to NK killing, but little immunogenic), and the speed of tumor growth. Thus, B16-F10 tumors grow out in 10–14 days, where 4T1 is a little slower taking approx. 21 days. As NK cells are part of the innate “immediate” response, the importance of this cell subset will be more visible in models of fast-growing tumors, e.g., B16-F10. Thus, it would be interesting to see if NK cells played a role at an early time point also against the 4T1 tumors. In addition when using nude mice we found that exercise reduced tumor size by approx. 60% compared to sedentary controls, but the mice had significantly larger tumors, than wild type C57Bl/6 mice [35]. This underscores an important role for T cells in tumor control, and thus T cells may be required for the therapeutic effect of exercise in long-term tumor models.

A recent study by Wennerberg and colleagues [45], who also used the 4T1 tumors, showed that exercise inhibited 4T1 breast tumor growth. However, no changes in the absolute number of infiltrating NK or T cells, but rather increased frequencies of proliferating effector NK and T cells, as well as a decreased frequency of immunosuppressive myeloid-derived suppressor cells (MDSC) was found in exercising animals. Improved responses were seen when focal radiotherapy and PD-1 blockade was combined with exercise. Strikingly, animals were only exercised one week subsequent to tumor inoculation, once a day for a limited period of 30 minutes.

The study by Wennerberg suggests that exercise modulates innate cells beyond NK cells. Indeed, we found increased numbers of dendritic cells in exercising animals [35], and exercise is known to mobilize a variety of innate cell types. A study by Almeida and colleagues [51] found decreased infiltration of tumor-associated macrophages and neutrophils in swiss mice injected with Ehrlich tumor cells, and an associated decrease in tumor growth. McClellan and colleagues [52] found that exercise decreases polyp formation in the Apc^Min+^ mouse model of intestinal tumorigenesis, which was associated with an overall decrease in expression of CCL22, a major chemoattractant for FoxP3^+^ Treg cells. Correspondingly, the authors found a decreased expression of FoxP3 and an increase in CD8 expression in exercising mice, compared to sedentary controls [52]. In a gene expression microarray analysis, we found that multiple chemokines, including CX3CL1 and CXCL10 which are associated with the attraction of antitumor NK and T cells, were increased in subcutaneous B16-F10 tumors from exercising mice [35]. Thus, it could be speculated that modulating the composition of macrophage numbers and phenotype, could alter the chemoattractant profile of the tumor microenvironment, promoting the infiltration of antitumor NK and T cells, and decreased attraction of immunosuppressive cell subsets such as MDSC and FoxP3^+^ Treg. 

**Figure 1:**
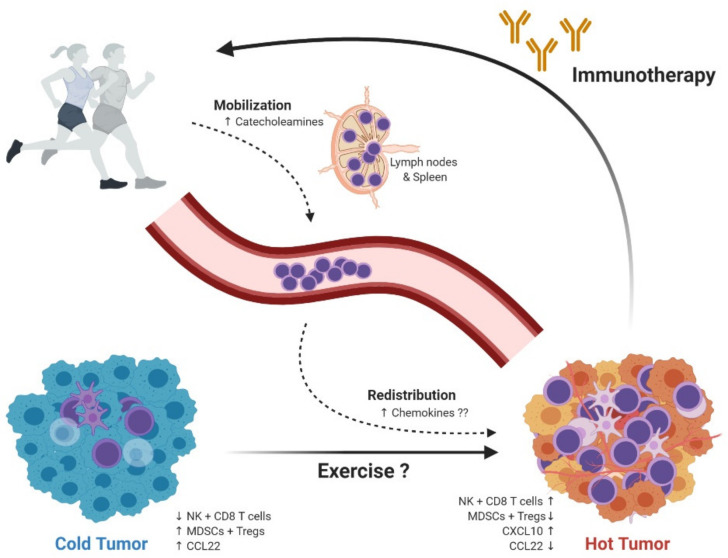
Exercise-dependent regulation of immune cells and modulation of the TME. Evidence from preclinical studies shows that exercise is associated with acute mobilization and redistribution of immune cells to tumors. This supports the potential role of exercise, as a modulator of the TME into a more antitumor environment. The hypothesis of exercise as a TME modulator is indicated in the figure, such as turning a cold tumor into a hot tumor; including higher infiltration of cytotoxic immune cells, decreased infiltration of suppressor immune cells, and altered chemokine expression. Finally, this modulation of TME into a more antitumor environment, suggests that exercise and immunotherapy could have a synergistic effect if combined. TME = tumor microenvironment. NK cells = natural killer cells. MDSCs = myeloid-derived suppressor cells. Tregs = regulatory T cells.

In summary, exercise mobilizes key effector cells of the immune system—T and NK cells—and studies from preclinical rodent models strongly suggest an anticancer effect largely mediated by mobilization of immune cells. Since these effector cells play key roles in anticancer responses in humans, this should set the stage for clinical testing (see overview in Figure 1).

## 5. Exercise Oncology; Focus on the Immune System; From Mouse to Man 

As in mice, exercise in humans can mobilize cells of the immune system. This phenomenon, exercise-induced leukocytosis, has been known for decades, and is in part due to increased cardiac output, blood flow and pressure, and associated shear stress [53]. Thus, exercise leads to a short-term activation of the biological stress response, resulting in the release of catecholamines, epinephrine, and norepinephrine to the bloodstream. As mentioned earlier, catecholamines bind to adrenergic receptors expressed by immune cells, causing the instant mobilization of those cells to the blood, increasing leucocyte numbers [54]. To this end, differentiated subsets of CD8 T cells, NK cells, and nonclassical monocytes are mobilized preferentially due to a high level of expression of the β2-adrenergic receptor [55]. The acute-stress-induced mobilization of lymphocytes leads to redistribution within different body compartments including areas of infections and tumors. Thus, the redistribution of lymphocytes has been shown to enhance the immune function in the skin, but it is still under debate if this occurs at all sites to which immune cells traffic during acute stress [54]. 

Additional aspects complicate matters further, e.g., whether and to which extent sex/gender and age influence the immune system, i.e., immune cell mobilization in response to exercise, and immune responses to cancer. Concerning sex, data are accumulating that checkpoint inhibitor treatment is less efficient in women, i.e., progression-free, as well as overall survival is greater in men than women [56]. Women have a higher risk of several autoimmune diseases, more or less pronounced depending on the disease [57]. Moreover, female rheumatoid arthritis patients respond less well to biologics, e.g., tumor necrosis factor-alpha inhibitors. On the other hand, females elicit more powerful antibody responses upon vaccination against viruses [58]. When it comes to physiological changes upon exercise very little is known, but differences in exercise response between men and women have been suggested by Timmons et al. [59]. Obviously, the topic needs further study.

Aging is associated with a progressing diminished functionality of the immune system termed immunosenescence [60]. Strikingly, immunosenescence is characterized by a decline in several immunological markers, a decline which occurs faster in males than females [61,62]. Some of these age-related changes in the immune system directly impact cell types that are key to the immunological response to exercise, NK, and T cell subsets, as mentioned also key cell types relevant for anticancer immune responses and response to CPI therapy. Obviously, this could raise concerns in terms of response to therapy. On the other hand, it offers the opportunity to see if markers of immune senescence decrease upon exercise, i.e., can immune senescence be reverted or delayed by exercise? Moreover, concerning the capacity to respond to immunotherapy in the elderly, data from small clinical trials suggested even better responses in this group of patients [63]. Still, data from large clinical studies are needed in order to elucidate if this is indeed the case [64]. 

Thus, exercise leads to a rapid increase in blood cell counts, and a rapid drop in the same counts after cessation of exercise. In fact, it was recently shown that cells egress the bloodstream minutes after exercise cessation directly correlated with the drop in heart rate [65]. Obviously, this very fast egress of mobilized cells from the blood, have implications in terms of when blood samples should be drawn, to allow evaluation of exercise-associated changes in immune cell subsets and functionality. 

Overall, acute exercise is now suggested as an important immune system adjuvant to stimulate the ongoing exchange of leukocytes between the circulation and tissues. However, exhaustive exercise and high-intensity training seen in, e.g., top athletics, has in contrast been linked to increased risk of illness [66]. This suggests an essential difference between acute-exercise-induced stress and activation of the immune system versus chronic-exercise-induced stress. Hence, a major ongoing focus is to investigate the exercise suitable for appropriate activation of the immune system in a cancer setting [67].

Beyond the above-mentioned effect on the immune system, exercise has a huge impact on the levels of several hormones and myokines. To the latter, contracting muscle secrete a very high number of myokines during exercise, which impacts the muscle cell itself but also on cells throughout the body. In terms of impact and potential relevance for cancer therapy, however, very little is known [68].

## 6. Exercise Oncology in the Clinic; Current Status and Future Prospects 

Historically, patients with cancer were recommended to rest and avoid strenuous activity following their cancer diagnosis, but this recommendation has changed dramatically in the last 40 years. A study conducted in 1978 by Lehmann et al, found that cancer was both associated with significant physical disabilities and negative psychosocial consequences for the patients [69]. These findings are reflected in the initial focus in early trials with cancer patients and exercise. A pioneer of randomized trials was conducted in the late 1980s, by Winningham and colleagues, where patients with breast cancer received exercise training as supportive care during chemotherapy [70]. This initial study, together with several others published over the following decades (summarized in a review by Cramp and Byron-Daniels from 2012 [71]), showed exercise as an effective supportive care strategy to improve the health-related quality of life (QoL), and counteract cancer-related fatigue (CRF). These initial findings changed the view of exercise and cancer and introduced a field of growing interest. 

A broader focus was initiated in the late 1990s, with the first trial by Dimeo and colleagues, showing that exercise had the potential of reducing chemo-related complications [72]. Patients with cancer of various origins were included in the study, but the majority were diagnosed with breast cancer (67%) [72]. This study provided new knowledge, by showing a lower risk of several chemotherapy-related complications, including shorter duration of neutropenia and thrombopenia; reduced severity of diarrhea and pain; and reduced length of hospitalization [72]. Expanding on these initial findings, Courneya and colleagues demonstrated in the START trial an improved treatment tolerance for patients doing resistance training. This randomized controlled trial (RCT) included 240 breast cancer patients, which at the eight-year follow up suggested a 25% reduced risk of death for the two training groups [73].

As given above, exercise has gained widespread use in clinical oncology, because it has been proven safe for cancer patients to exercise, shown improvement of quality of life, maintained muscle strength during therapy, and reduced treatment-related complication [74,75]. In terms of exercise in relation to cancer risk, a recent study pooled data from 12 prospective studies including 1.44 million adults (over 180,000 cancer patients), and showed that physical activity lowered the risk for many cancers [76]. Concerning disease outcome, it has been shown that physical activity reduced all-cause but also cancer-specific death in patients with breast and colon cancer [77]. Similar data have been suggested in prostate cancer [78,79]; Interestingly, a small RCT study by Hvid et al. (including 25 patients), found a significant increase in PSA doubling time, for patients undertaking a two years home-based endurance training program [80]. 

Obviously, these data on disease outcome, although interesting, reveal little information as to the mechanism of action. Thus, despite the very encouraging data, major challenges in the field of exercise oncology have also emerged. Several publications are highlighting the need for large scale randomized controlled trial (RCT) to compare exercise modalities, ranging from aerobic to resistance exercise training, intensity, duration, supervision, predictive biomarker, etc., and to which extent these can improve clinical outcomes [81]. Therefore, it is encouraging and will be interesting to see the results from large RCT such as CHALLENGE (NCT00819208) including patients with colon cancer and INTERVAL (NCT02730338) including patients with prostate cancer. Both studies are multinational and multicenter studies aiming at 850–1000 patients included and have overall survival and disease-free survival as primary outcomes [82,83,84]. In general, exercise oncology is subject to growing interest in the field and the number of trials registered at clinicaltrial.gov has exploded [67]. A key prerequisite for these trials to lead to true benefit in terms of improved treatment regimens is a better understanding of the underlying mechanisms for how exercise may impact cancer progression and survival. Most of our knowledge of mechanisms comes from studies in rodents or from in vitro studies, both of which have provided some encouraging results (as described above). The next step would be to test these preclinical results in a clinical setting (see overview in Figure 1).

## 7. Exercise Oncology in the Clinic; HI AIM

In the last 10 years, the introduction of immunotherapy for the treatment of patients with NSCLC has transformed the therapeutic landscape. Impressive results using checkpoint inhibitors, especially PD1 as monotherapy or in combination with chemotherapy, have improved the overall survival of NSCLC patients [85,86]. As the field has developed, the TME has become a major focus for determining a suitable treatment regime for patients with NSCLC. At the moment, the percentage of tumoral PDL1 expression (defined as PDL1 expression being <1%, 1–49%, and ≥50%), is being used as a marker to determine treatment strategy [87,88]. Nonetheless, as discussed earlier, the TME is complex and highly dynamic, and since PDL1 is upregulated by IFNγ, the influx of immune cells that secrete IFNγ upon activation in the TME, may lead to upregulation of PDL1 [89]. Numbers of neoantigens and presence of high numbers of T cells has also been correlated with response, but remain too weak, and too complicated, to be used as predictive markers [90]. Despite this, it does underscore the importance of improving the influx of immune cells to the TME.

Two important aspects of checkpoint inhibitor therapy should be mentioned. First, there is, for many good reasons, a focus on T cells as main effector cells, being accountable for the response to therapy based on the PD1/PDL1 breach. In turn, this implies that the true target during therapy is in fact a class I HLA molecule expressed by cancer cells. Second, it has been shown that immunotherapy may lead to the selection of escape variants; i.e., the outgrowth of cancer cells with defects in downstream IFNγ signaling [91,92], or with genetic hits in HLA expression [92,93,94]. Overall, supporting strong evidence for T cells being prominent effector cells during therapy. However, it has also been shown that HLA loss can frequently be detected in NSLC even before immunotherapy [95,96], pointing out that immunoediting can take place during the natural immune response elicited in patients. This underscores the potential importance of considering effector cells beyond CD8 T cells. Exercise mobilizes most pronouncedly NK cells, but also unconventional T cells; e.g., γδ T cells and NKT cells [55]. These cell types are capable of killing HLA negative cancer cells and could be important for response rate and duration. Thus, introducing exercise to patients with NSCLC could test if exercise can be used as a tool to modulate the TME into a more antitumor environment, based on the infiltration of different types of immune cells with antitumor function.

Therefore, in the fall of 2020, we will initiate a clinical trial entitled: High-Intensity Aerobic exercise training and Immune cell Mobilization in patients with lung cancer (HI AIM) (NCT04263467). The overall purpose of this study is to investigate if medium to high-intensive training can mobilize and activate the immune system, and thereby enhance the effect of the conventional treatment of lung cancer patients. As an important aspect of this study, we will investigate if the presence of various proteins and immune cells in blood and tumor biopsies, can verify or predict the effect of the high-intensity training.

The study will be a randomized controlled trial (RTC), including 70 patients with NSCLC. Concurrent with the exercise intervention, all patients will receive standard oncological treatment, being: checkpoint inhibitors (anti-PD1), checkpoint inhibitors (anti-PD1) combined with chemotherapy, or oncological surveillance. Patients will be stratified according to their treatment regime, following randomization into either the intervention or control group. Patients in the intervention group will receive a six-week exercise-based training, which will be group-based and supervised by a physiotherapist. Each training session will last approximately 40 minutes and consist of intermediate and high-intensity interval training. Included patients will be between 18 and 70 years old, but there will be no gender or ethnicity restrictions for entering the trial. 

To monitor the response of the trial, different analysis tools will be applied, including questionnaires (QNRs) covering the patient’s quality of life (QoL) and physical activity at baseline, during, and at trial completion. All patients will also have their VO_2_ peak tested at baseline and again after the exercise intervention. The purpose of the QNRs and VO_2_ peak tests, is to control and verify the effect of exercise training of the intervention group and to monitor the control group. 

The primary endpoint will be to monitor the effect of exercise on circulating immune cells. To do so, several blood samples will be taken at baseline, during, and at trial completion. In addition, tumor biopsies will be taken at baseline and again after the exercise intervention, if patients have metastasis allowing for this procedure. This will allow us to do several immunological analyses (including flow cytometry, sequencing, ELISA/Luminex) focusing on mobilization and redistribution of immune cells in response to exercise. Finally, all data will be correlated to clinical data such as overall survival and progression-free survival. The present randomized controlled study will, thereby, generate new knowledge about how exercise impacts the immune systems for patients with NSCLC.

## 8. Conclusions

Immunotherapy of cancer and in particular the use of monoclonal antibodies that block checkpoint inhibitory signaling in cells of the immune system has revolutionized the treatment of a range of malignancies. Data from preclinical studies in rodents support exercise-dependent mobilization and redistribution of immune cells to tumors. These data support the potential role of exercise, as an indirect modulator of the TME into a more antitumor environment. Clinical and epidemiological studies also provide evidence that exercise is safe, feasible, and beneficial for cancer patients. Therefore, RCTs testing the combination of exercise training and immunotherapy for patients with cancer is very timely and may act to increase the effectiveness of immunotherapy. Given the complexity of the immune system and anticancer immune responses, clinical trials should focus on increasing our understanding of the underlying mechanisms. This is essential because the elucidating mechanism of how exercise impacts human cancer is beneficial for designing future more efficacious treatments and, thus, is important for the entire field of oncology.
